# Postoperative outcomes after total sevoflurane inhalation sedation using a disposable delivery system (Sedaconda-ACD) in cardiac surgery

**DOI:** 10.3389/fmed.2024.1340119

**Published:** 2024-03-05

**Authors:** François Labaste, Paul Cauquil, Magda Lestarquit, Pascale Sanchez-Verlaan, Abdulrahman Aljuayli, Bertrand Marcheix, Thomas Geeraerts, Fabrice Ferre, Fanny Vardon-Bounes, Vincent Minville

**Affiliations:** ^1^Anesthesiology and Critical Care Unit, Toulouse University Teaching Hospital, Toulouse, France; ^2^RESTORE, UMR 1301 Inserm - 5070 CNRS - Université Paul Sabatier, Université de Toulouse, Toulouse, France; ^3^Department of Cardiac Surgery, Toulouse University Teaching Hospital, Toulouse, France

**Keywords:** sevoflurane, cardiovascular surgery, troponin, Sedaconda, cardiac surgery

## Abstract

**Introduction:**

The COVID-19 pandemic prompted our team to develop new solutions for performing cardiac surgery without intravenous anesthetics due to a shortage of these drugs. We utilized an anesthetic conserving device (Sedaconda-ACD) to administer total inhaled anesthesia because specific vaporizers were unavailable for administering inhaled agents during cardiopulmonary bypass (CPB) in our center. We documented our experience and postoperative cardiovascular outcomes. The primary outcome was the peak level of troponin, with secondary outcomes encompassing other cardiovascular complications.

**Material and methods:**

A single-center retrospective study was conducted. We performed a multivariate analysis with a propensity score. This investigation took place at a large university referral center.

**Participants:**

Adult patients (age ≥ 18) who underwent elective cardiac surgery with CPB between June 2020 to March 2021.

**Intervention:**

During the inclusion period, two anesthesia protocols for the maintenance of anesthesia coexisted—total inhaled anesthesia with Sedaconda-ACD and our classic protocol with intravenous drugs during and after CPB.

**Primary endpoint:**

Troponin peak level recorded after surgery (highest level recorded within 48 h following the surgery).

**Results:**

Out of the 654 included patients, 454 were analyzed after matching (intravenous group = 297 and inhaled group = 157). No significant difference was found between the groups in postoperative troponin peak levels (723 ng/l vs. 993 ng/l–*p* = 0.2). Total inhaled anesthesia was associated with a decreased requirement for inotropic medications (OR = 0.53, 95% CI 0.29–0.99, *p* = 0.04).

**Conclusion:**

In our cohort, the Sedaconda-ACD device enabled us to achieve anesthesia without intravenous agents, and we did not observe any increase in postoperative complications. Total inhaled anesthesia with sevoflurane was not associated with a lower incidence of myocardial injury assessed by the postoperative troponin peak level. However, in our cohort, the use of inotropic drugs was lower.

## 1 Introduction

Anesthetic management of patients requiring cardiopulmonary bypass (CPB) for cardiovascular surgery has been studied extensively but remains largely controversial. Many studies have shown that the use of volatile anesthetics, such as sevoflurane, can mimic the early phase of ischemic preconditioning through multi-pathway signaling of mitochondrial KATP channels (1). Moreover, there is some evidence that late post-conditioning with the volatile anesthetic sevoflurane might confer cardiac protection (2). While there is growing evidence of the cardio-protective effects of volatile anesthetics in animals, studies in humans yield conflicting results. Several studies have suggested that the use of volatile anesthetics during cardiac surgery, especially during coronary artery bypass grafting (CABG), could enhance myocardial protection and reduce the risk of perioperative myocardial infarction and myocardial dysfunction (3–6). However, other randomized clinical trials have not confirmed the benefit of volatile anesthetics (7, 8). A recent pragmatic multicenter controlled trial, which aimed to replicate real-life conditions, showed that, among patients undergoing elective CABG, anesthesia with a volatile agent did not result in significantly fewer deaths in 1 year than total intravenous anesthetics (9). Therefore, there are no strong recommendations for sedation use in cardiac surgery, whether by intravenous or inhaled agents. International guidelines suggest that the data are not conclusive enough to recommend a particular modality of anesthesia for cardiac surgery (10, 11). Therefore, several centers, including ours, perform mixed anesthesia—inhaled sedation before CPB followed by intravenous sedation during CPB and intensive care unit (ICU).

The COVID-19 pandemic led to a high consumption of intravenous sedative drugs worldwide, thereby resulting in a shortage of their supply. Anesthetic agents, such as propofol or midazolam, made their appearance on the FDA's shortage list (12). Continued cardiac surgery activities, especially in centers that usually maintain intravenous anesthesia as a main strategy, may have been problematic with the issue of a shortage of supply. Our center was particularly affected. Specifically, we ran out of propofol to sedate the patients. Additionally, we did not have dedicated vaporizers for CPB to administer sevoflurane directly to the oxygenator. We considered the idea of diverting the usual use of an anesthesia preservation device to enable us to provide anesthesia with total inhalation anesthesia with sevoflurane. We have previously described our solution to perform cardiac surgery without intravenous sedative anesthetic drugs by using an anesthetic conserving device (Sedaconda ACD-S, Sedana Medical, Uppsala, Sweden) (13). Sedaconda-ACD appears to be a practical and good device for providing total inhaled anesthesia with sevoflurane.

In this study, we present our postoperative results following the use of total inhaled anesthesia with sevoflurane during the pandemic. Furthermore, based on previously published data regarding the cardiovascular effects of sevoflurane, we hypothesized that total inhaled anesthesia with sevoflurane might improve postoperative cardiovascular outcomes.

## 2 Materials and methods

### 2.1 Patients

We performed a retrospective, single-center, observational study between June 2020 and March 2021 at Toulouse University Hospital (Toulouse, France).

According to French laws on ethics, patients were informed that their codified data would be used for the study. According to the French ethics and regulatory law (public health code), retrospective studies based on the exploitation of usual care data need not be submitted to an ethics committee, but they must be declared or covered by reference methodology of the French National Commission for Informatics and Liberties (CNIL). A collection and computer processing of personal and medical data was implemented to analyze the results of the research. Toulouse University Hospital signed a commitment of compliance to the reference methodology MR-004 of CNIL. After evaluation and validation by the data protection officer and according to the General Data Protection Regulation, this study completed all the criteria, and it was registered in the register of retrospective studies of the Toulouse University Hospital (register number: RnIPH 2020-124) and covered by MR-004 (CNIL number: 2206723 v 0).

All consecutive patients admitted to the cardiovascular surgery unit for elective cardiac surgery with the use of CPB were included. The inclusion criteria were age ≥ 18 years and elective cardiac surgery with the use of CPB; the exclusion criteria were emergency surgery, surgeries without CPB, heart transplantations, and surgeries for left ventricular assist device implantations.

### 2.2 Perioperative management

During the study period and in response to the shortage of intravenous anesthesia medications (propofol), we developed two anesthesia protocols that coexisted.

The intravenous protocol was the standard protocol used in our center before the pandemic. Anesthesia was induced with sufentanil and propofol and maintained by a mixed use of intravenous and inhaled agents. The volatile agent, sevoflurane, was used between the induction of anesthesia and the initiation of CPB. The intravenous agent, propofol, was used during CPB and continued until the patient was ready for wake-up after surgery in the ICU.

The inhaled protocol was implemented during the COVID-19 pandemic. Our solution has been previously described and was based on the utilization of a small Sedaconda-ACD device, with a dead space of 50 ml (Anaesthetic Conserving Device, Sedana Medical, Uppsala, Sweden) (13). After anesthesia induction, Sedaconda-ACD was inserted in the breathing circuit between the endotracheal tube and the Y-piece. At the beginning of the CPB, the Sedaconda-ACD was placed just before the oxygenator on the oxygen tube. Sevoflurane was used. After the removal of CPB, Sedaconda-ACD was moved back to the breathing circuit. At the end of the surgery, for postoperative sedation in the ICU, the Sedaconda-ACD was kept on the patient, and volatile infusion was continued until the decision was made to wake up the patient. Throughout the perioperative period, sedation was adjusted to target a minimum alveolar concentration (MAC) ranging between 0.8 and 1.2.

The choice between the two protocols was guided by the availability of anesthetic drugs.

All other anesthetic and surgical procedures were standardized.

Sufentanil was used for analgesia, and neuromuscular blockade with atracurium was performed before intubation. Additional sufentanil boluses were administered during surgery, and continuous atracurium was maintained. The depth of sedation was monitored using the Bispectral Index (BIS–Medtronic^®^), and values were maintained within the range of 40–60.

CPB was standardized with a target blood flow of 2.5 L/min per m^2^. The pump prime for the CPB circuit contained 1,200 mL of crystalloids (Ringer-Lactate; Viaflo). After systemic heparinization to achieve an activated clotting time (ACT) level of 450 s, median sternotomy and central cannulation (aortic and right atrial) were performed. Myocardial protection was initially ensured with antegrade cold blood cardioplegia and then maintained either with retrograde cardioplegia or by intermittent antegrade cardioplegia. Throughout the entire surgery, normothermia was maintained. Normoglycemia (arterial blood glycemia between 5.5 and 11 mmol/l) was maintained with intravenous bolus insulin, if necessary.

The mean arterial pressure target was 65 mmHg, and norepinephrine was administered to achieve that objective. If necessary, fluid administration with crystalloids was given. At the end of the procedure, in case of complex CPB weaning, a positive inotropic agent was administered to the patient. Initiation of an inotropic drug occurred after confirmation of left and/or right ventricular dysfunction on transesophageal echocardiography. Patients with a hemoglobin value below 8 g/dL received homologous red blood cell transfusions.

After surgery, patients were admitted to the surgical ICU. Anesthesia was continued until the patient was ready for wake-up. Patients were withdrawn from anesthesia once they achieved satisfactory hemodynamic and respiratory stability, normothermia, and the absence of significant hemorrhage (< 0.5 mL/kg per hour), all of which were carried out following a standardized protocol. Circulatory drug support was guided by our institutional protocol and was standardized.

### 2.3 Data collection

We collected data on baseline characteristics and coexisting conditions, intraoperative care, postoperative duration of stay in an intensive care unit and in the hospital, major outcomes, and adverse events. The following variables were recorded: age, gender, body weight, height, personal medical history, ASA score, EuroSCORE II, type of cardiac surgery, preoperative left ventricular ejection fraction, the duration of CPB, the duration of aortic clamping, the need for intraoperative blood transfusion, the need for inotropic support, time to extubation, and any complications that occurred during the surgery and/or in the ICU. The degree of myocardial injury was assessed using troponin I levels. Postoperatively, troponin levels were collected upon ICU admission at 6:00 a.m. on day 1 and day 2 and whenever it was judged to be necessary by the responsible physician.

### 2.4 Endpoints

Our objective was to describe the postoperative outcomes of patients by presenting postoperative complications, with a particular focus on describing the cardiovascular outcomes.

The primary endpoint was the troponin peak level recorded after surgery. We selected the highest level recorded within the initial 48 h following the surgery.

The secondary endpoints were the need and duration of catecholamine infusion (vasopressors and positive inotropes), the need for temporary cardiac assistance such as extracorporeal life support (ECLS), supraventricular arrhythmias such as atrial fibrillation or flutter during ICU stay, duration of mechanical ventilation, incidence of re-intubation episodes, postoperative delirium diagnosed with the CAM-ICU (confusion assessment method, ICU version) during the ICU stay, acute kidney injury (using KDIGO criteria, KDIGO ≥2), and the length of intensive care unit and hospital stay.

### 2.5 Statistical analysis

We calculated that 191 patients per group would allow us to demonstrate a reduction of 300 ng/ml of the postoperative troponin level peak, with a power of 0.9 and an alpha risk of 0.05. This hypothesis was based on a previous study which had similar objectives (9, 14).

The normality of the data distribution was assessed using the Shapiro–Wilk test. Continuous variables were summarized as means (± standard deviation) and compared using the Mann–Whitney test. Categorical variables were summarized as counts and percentages and compared using Pearson's chi-squared test.

We conducted a multivariable logistic regression analysis with propensity score matching, which was defined as the probability of exposure to total inhaled anesthesia (15). We selected only the covariates most likely to introduce a confounding bias based on clinical expertise and inputs from the literature. These covariates were EuroSCORE II, CPB time, and red blood transfusion. In addition, it was planned to include the preoperative data that appeared to be statistically different between the two groups in the propensity score. Therefore, we selected the variable body mass index. Next, we performed matching with replacement between patients from the total inhaled anesthesia and those from the mixed anesthesia group in a 1:2 ratio (propensity score matching, optimal model, Mahalanobis distance matching). Acceptable covariate balance was defined as a standardized mean difference of < 0.1 for the entire list of covariates. Finally, we undertook multivariate weighted logistic regression with troponin as an outcome variable and the treatment group and the matched variables as explanatory variables.

To investigate risk factors for the use of inotrope drugs, we divided our population into two groups (with or without the use of inotrope). We performed a univariate analysis as described above. Then, we performed a multivariate analysis (logistic regression) by estimated odds ratios. We used a top-down stepwise procedure (backward elimination) that consisted of including all variables with a *p*-value of < 0.2 and then progressively removing the non-significant ones. A *p-*value of < 0.05 was considered statistically significant.

All analyses were conducted using XLSTAT (Addinsoft, version 2019.1.1.62918) and RStudio (Version 2023.12.0+369 (2023.12.0+369).

## 3 Results

From June 2020 to March 2021, we screened 775 patients for eligibility. A total of 654 patients were included in this study, and 121 patients were excluded due to missing data. All excluded patients had received intravenous anesthesia. A total of 170 patients (26%) underwent total inhaled anesthesia with sevoflurane using Sedaconda. The baseline characteristics are presented in Table 1. The mean age was 65.5 (±11.2) years, and the mean EuroSCORE II was 5.6 (±6.4). Postoperative outcomes before matching are detailed in Table 2.

**Table 1:** Baseline characteristics.

	**Total *N* = 654**	**Intravenous anesthesia *N* = 484**	**Inhaled anesthesia *N* = 170**	***p***
**Preoperative data**				
Age, years	65.5 (11.2)	65.2 (11.8)	66.7 (9)	0.46
Gender, male % (n)	72.3 (473)	72.9 (353)	70.6 (120)	0.56
Weight, kg	78.4 (15.2)	77.8 (15.3)	80.1 (14.8)	**0.04**
Size, m	1.7 (0.09)	1.7 (0.09)	1.69 (0.08)	0.26
BMI, kg/m^2^	27.1 (4.7)	26.8 (4.7)	27.8 (4.42)	**0.005** ^**#**^
EuroSCORE II	5.6 (6.4)	5.9 (6.9)	4.4 (4.3)	**0.008** ^**#**^
Ischemic heart disease, % (n)	48.2 (315)	47.5 (230)	50 (85)	0.58
Previous cardiac surgery, % (n)	6.7 (44)	8.1 (39)	2.9 (5)	**0.02**
Chronic arterial hypertension, % (n)	55.7 (364)	55.2 (267)	57.1 (97)	0.67
Smoke, % (n)	2.2 (15)	1.9 (9)	3.5 (6)	0.33
Chronic occlusive arteriopathy, % (n)	7.5 (49)	8.8 (43)	3.5 (6)	**0.02**
Diabetes (type 1 or 2), % (n)	26 (170)	24 (116)	31.8 (54)	**0.05**
COPD, % (n)	16.7 (109)	17.3 (84)	14.7 (25)	0.43
Stroke, % (n)	7.3 (48)	7.6 (37)	6.4 (11)	0.61
Left ventricular ejection fraction, %	57.6 (10.9)	57.2 (11.1)	58.8 (9.9)	0.32
Chronic renal insufficiency, % (n)	16.1 (105)	17.6 (85)	11.6 (20)	0.08
eGFR, ml/min/1,73 m^2^	77.6 (22.2)	77.1 (23.1)	79.2 (19.4)	0.43
Preoperative Hemoglobin, g/dl	13.6 (1.7)	13.6 (1.8)	13.8 (1.5)	0.17
**Perioperative data**				
Type of surgery				0.78
Ascendant aortic surgery, % (n)	12.5 (82)	13.4 (65)	10.0 (7)	
Aortic valve surgery, % (n)	18.8 (122)	17.7 (85)	21.2 (36)	
Mitral valve surgery, % (n)	11.6 (76)	12.2 (59)	10 (7)	
CABG, % (n)	44.8 (293)	43.8 (212)	47.6 (81)	
Valve surgery + CABP, % (n)	8.4 (55)	8.5 (41)	8.2 (14)	
Aortic valve + mitral valve, % (n)	2.1 (14)	2.3 (11)	1.8 (3)	
Other, % (n)	1.8 (12)	2.1 (10)	1.2 (2)	
Complex surgery, % (n)	38.7 (253)	40.7 (197)	32.9 (56)	0.07
Atrial fibrillation ablation, % (n)	8.4 (55)	8.7 (42)	7.6 (13)	0.68
Ascending aorta, % (n)	15 (98)	15.7 (76)	13.9 (22)	0.39
Surgery time, min	239 (71)	239 (72)	238 (67)	0.69
CPB time, min	90 (39)	92 (41)	85 (34)	0.06^#^
Aortic cross clamp time, min	64.5 (30.1)	65.7 (31.2)	61 (26.7)	0.13
Transfusion, % (n)	8.1 (53)	10.1 (49)	2.3 (4)	**0.001** ^**#**^

Quantitative data: mean (standard deviation). Qualitative data: frequency in % (number of subjects). BMI, Body Mass Index; COPD, Chronic Obstructive Pulmonary Disease; Chronic Renal Failure (GFR < 60 ml/min/1.73 m^2^); GFR, Glomerular Filtration Rate; CPB, cardiopulmonary bypass; CABG, coronary artery bypass grafting. ^#^Data included in the propensity score. Bold values indicate statistical significance.

**Table 2:** Postoperative outcomes before matching.

	**Total *N* = 654**	**Intravenous anesthesia *N* = 484**	**Inhaled anesthesia *N* = 170**	***p***
**Quantitative data**				
Troponin HS, ng/l	968 (1,986)	1,056 (2,231)	715 (885)	**0.002** ^*^
Lactate Day 1	2.4 (1.8)	2.5 (1.9)	2.4 (1.9)	0.52
Intubation duration	1.6 (3.1)	1.7 (3.4)	1.5 (2.4)	0.08
ICU length of stay	5.4 (4.9)	5.5 (5.1)	5.15 (4.3)	0.2
Hospitalization Length of stay	11.9 (6.5)	12 (6.6)	11.7 (6.5)	0.19
SAPS 2	32.9 (9.9)	33.2 (10.3)	32.2 (8.6)	0.41
**Qualitative data**				
Vasopressor	93,2 (610)	92.1 (446)	96.5 (164)	**0.05** ^*^
Inotrope	15.9 (104)	18.6 (90)	8.2 (14)	**0.001** ^*^
Re-intubation	5.8 (38)	6.4 (31)	4.1 (7)	0.27
Atrial fibrillation	38.8 (254)	39 (189)	38.2 (65)	0.85
Acute renal failure	34.3 (224)	36.6 (177)	27.6 (47)	**0.03** ^*^
Delirium	93,2 (610)	92.1 (446)	96.5 (164)	**0.05** ^*^
Hospital death	15.9 (104)	18.6 (90)	8.2 (14)	**0.001** ^*^

Quantitative data: mean (standard deviation). Qualitative data: frequency in % (number of subjects). Times are expressed in days. Troponin HS, hypersensitive troponin in ng/l; SAPS2, Simplified Acute Physiology Score. ^*^Statistically different data, *p* < 0.05. Bold values indicate statistical significance.

### 3.1 Total inhaled anesthesia with sevoflurane did not reduce the postoperative troponin peak level

Although the postoperative troponin peak level recorded during the first 48 postoperative h was significantly lower in the total inhaled anesthesia with sevoflurane, after matching, the difference was not statistically significant (723 ng/l vs. 993 ng/l–*p* = 0.2) (Table 3).

**Table 3:** Postoperative outcomes after matching.

	**Intravenous anesthesia *N* = 297**	**Inhaled anesthesia *N* = 157**	***p***
**Quantitative data**			
Troponin HS, ng/l	993 (2,035)	723 (889)	0.2
Lactates Day 1	0.49 (1.38)	0.27 (0.97)	**0.05** ^*^
Intubation duration	2.53 (1.98)	2.35 (1.37)	0.99
ICU length of stay	2.2 (2.2)	2.3 (1.8)	0.14
Hospitalization Length of stay	1.7 (3.8)	1.5 (2.2)	0.31
SAPS 2	5.3 (5.4)	5 (4.1)	0.52
**Qualitative data**			
Vasopressor	89.9 (267)	96.8 (152)	**0.009** ^*^
Inotrope	15.5 (46)	8.9 (14)	**0.04** ^*^
Re-intubation	(7.1 521)	3.8 (6)	0.16
Atrial fibrillation	39.1 (116)	37.6 (59)	0.76
Acute renal failure	34.3 (102)	28.2 (44)	0.17
Delirium	10.1 (30)	13.4 (21)	0.29
Hospital death	4 (12)	1.3 (2)	0.1

Quantitative data: mean (standard deviation). Qualitative data: frequency in % (number of subjects). Times are expressed in days. HS, hypersensitive troponin in ng/l; SAPS2, Simplified Acute Physiology Score2. ^*^Statistically different data, *p* < 0.05. Bold values indicate statistical significance.

### 3.2 Total inhaled anesthesia with sevoflurane reduced the use of positive inotrope

In our univariate analysis, the percentage of patients requiring post-CPB use of inotropic catecholamine was significantly lower in the total inhaled anesthesia with the sevoflurane group using Sedaconda (8.2% vs. 18.6%–*p* = 0.002). After matching, the difference remained significant, with an OR of 0.53 (95% CI: 0.29–0.99; *p* = 0.04) (Table 3).

However, after matching, when inotropic catecholamines were used, the duration of infusion was the same in both groups (3.2 +/– 2.0 IV group vs. 3.1 +/– 1.4 inhaled group, *p* = 0.99).

We analyzed the risk factors for the use of a positive inotrope in our cohort. The results of the univariate analysis are presented in Supplementary material. After the multivariate analysis, among the independent risk factors for the use of a positive inotrope in the postoperative period, we found total inhaled anesthesia with sevoflurane using Sedaconda as a protective factor (OR = 0.51; IC95% 0.27–0.97; *p* = 0.04) (Table 4).

**Table 4:** Risk factors of postoperative inotrope administration (multivariate analysis).

	**OR**	**IC (95%)**	***p***
EuroSCORE II	1.03	0.99–1.07	0.054
LVEF, %	0.95	0.93–0.97	< 0.0001
CPB, minute	1.01	1.0–1.01	0.002
Complex surgery	2.07	1.2–3.49	0.007
Chronic arterial hypertension	1.72	1.05–2.82	0.03
Inhaled anesthesia with sevoflurane	0.51	0.27–0.97	0.04
Chronic renal failure	2.13	1.21–3.73	0.008

Hosmer-Lemeshow test: 0.09. Aera under the ROC Curve (AUC) of model: 0.79; LVEF, Left ventricular ejection fraction; ECG, Extracorporeal circulation; AH, Arterial hypertension; Chronic renal failure with glomerular filtration rate < 60 ml/min/1.73m^2^.

### 3.3 Total inhaled anesthesia with anesthesia increased the risk of using vasopressor

In our univariate analysis, the percentage of patients requiring post-CPB use of vasopressor was significantly lower in the inhaled anesthesia group (8.2% vs. 18.6%–*p* = 0.002). After matching, the difference remained significant, with an OR of 0.53 (95% CI: 0.29–0.99; *p* = 0.04) (Table 3).

However, after matching, when inotropic catecholamines were used, the duration of infusion was the same in both groups (2.6 +/– 2.1 IV group vs. 2.4 +/– 1.9 inhaled group, *p* = 0.37).

### 3.4 The use of total inhaled anesthesia with sevoflurane using Sedaconda did not increase the incidence of postoperative complications

In our cohort, the use of total inhaled anesthesia with sevoflurane using Sedaconda did not increase the length of stay in the intensive care unit or the length of hospital stay (Table 2). Moreover, no patient in this cohort required mechanical cardiac assistance, such as ECLS, during the postoperative period.

The incidence of postoperative acute renal failure was lower in the inhaled anesthesia group than in the intravenous anesthesia group (27.6% vs. 36.6%; *p* = 0.03). However, after matching, inhaled anesthesia was not a significant protective factor for the risk of acute renal failure (OR = 0.74; 95% CI 0.49–1.14; *p* = 0.17) (Table 3).

There were no differences in the incidence of postoperative delirium episodes or the incidence of re-intubation between the two groups (Table 2).

## 4 Discussion

In this study, maintaining anesthesia with sevoflurane using a disposable delivery system (Sedaconda-ACD) was not associated with an increase in postoperative complications. We did not observe a reduction in postoperative troponin peak levels. However, the total inhaled anesthesia was linked to a decreased need for positive inotropic agents in the postoperative period.

The COVID-19 pandemic prompted us to innovate, enabling our institution to sustain cardiac surgery activities, and we diversified the use of Sedaconda (13). Total inhaled anesthesia with sevoflurane is rarely employed in adults. This is largely attributed to the challenges associated with administering volatile anesthetics during CPB and in the postoperative period in the ICU. The administration of volatile agents during and after CPB involves the use of different devices. The use of volatile agents on the CPB oxygenator necessitates specialized vaporizers, which are not universally available, particularly in European centers. Therefore, the use of the AnaConDA™ device presents a novel solution for facilitating the continuous administration of sevoflurane during cardiac surgery, including CPB and ICU sedation.

The utilization of the Sedaconda device, enabling the continuous administration of volatile agents, appears particularly relevant to ensure the full expression of effects on pre- and post-conditioning. The ischemia preconditioning effects of inhaled agents have been extensively studied in the literature. Notably, a recent randomized trial reported that inhaled agents did not have a positive impact on the postoperative course of coronary artery bypass graft surgery (9). The objective of this study was to adopt a pragmatic approach to the administration of inhaled agents in cardiac surgery. However, due to the absence of a standardized protocol for the administration of inhaled and intravenous agents, a limitation of this study was that total inhaled anesthesia was not consistently administered, especially since it was disrupted during CPB and the postoperative period. Despite the absence of a significant difference in troponin peak levels, the lack of maintaining sevoflurane anesthesia during parts of the procedure introduced a notable bias in this study (9, 16). Additionally, when propofol is administered during inhaled anesthesia, co-administration has been shown to attenuate the potential beneficial effects of volatile anesthetics (9, 17).

However, in our study, outcomes regarding the cardioprotective properties of total inhaled anesthesia are mixed.

On the one hand, we were not able to demonstrate any benefit in reducing myocardial ischemic lesions, as the postoperative troponin peak level was not decreased. This result is consistent with other studies in which sevoflurane was administered intraoperatively and postoperatively (14, 18, 19). Several factors may explain this result. First, we did not standardize the administration of sevoflurane to achieve cardioprotection. In our unit, the adoption of inhaled anesthesia intra operatively and postoperatively, along with the use of Sedaconda, became necessary due to the shortage of intravenous anesthesia drugs during the COVID-19 pandemic (13). Specifically, we did not set MAC targets but only sedation goals, maintaining a BIS^®^ (Bispectral index) between 40 and 60. The cardioprotective effect of sevoflurane appears to be more pronounced when MAC is >1.2 (20). Given the retrospective design of our study, reporting that the levels of MAC intraoperatively and postoperatively was not feasible, we were unable to incorporate these data into the interpretation of our results. Additionally, other factors such as intraoperative lidocaine administration and intraoperative and postoperative glycemic control are known to influence the protective effect of sevoflurane (14, 21). In our cohort, all patients received lidocaine, potentially impacting the results. Glycemic management was protocolized, and patients receiving insulin were evenly distributed between the two groups. While older age could be a factor attenuating the preconditioning effect, there was no significant difference in age between the two groups (22).

On the other hand, we demonstrated that the administration of sevoflurane appeared to exhibit cardioprotective properties. Inhaled anesthesia emerged as an independent protective factor against the risk of using positive inotropic catecholamines. To date, few studies have reported the effect of intraoperative and postoperative maintenance with inhaled anesthesia on myocardial function (14, 19). In these randomized studies, the primary objective did not focus on the use of inotropes or myocardial function. Guinot et al. (14) reported that positive inotropes were administered in only 5% of cases. The study's sample size (81 patients) was insufficient to demonstrate an effect on the use of inotropes (14).

However, it appears that cardiac function after bypass surgery is improved in the presence of halogenated agents. Wasowicz et al. reported a higher post-CPB cardiac output in the presence of halogen agents when compared with intravenous agents (19). The results of our work seem to confirm these findings. Thus, in our cohort, while not significantly impacting biomarkers of myocardial ischemia, sevoflurane has demonstrated its ability to protect the myocardium by reducing the need for positive inotropes in the postoperative period. The clinical implications of this result reopen the debate on the value of halogenated agents. Indeed, troponin appears to be a weakly relevant marker for studying the cardioprotective effects of one strategy vs. another.

The assessment of postoperative troponin levels is a classic endpoint to describe the intensity of ischemic injury. However, this description is not sufficient as it does not account for the impact of the injury on cardiovascular function (23). Indeed, the use of inotropes appears to be strongly associated with poor postoperative outcomes, making the development of strategies to limit their use particularly important (24).

We found a higher proportion of patients requiring vasopressors, but the duration of infusion of these drugs was not significantly different between the two groups. The exclusive use of inhaled agents for the maintenance of anesthesia was associated with an increased need for vasoactive catecholamines, confirming data from previous studies (14, 18). Sevoflurane is known to have a dose-dependent impact on macrohemodynamics and microhemodynamics, with a vasodilatory effect that is more pronounced than certain intravenous agents, such as propofol (14, 19). The occurrence of endothelial dysfunction, classically observed after cardiac surgery, could be favored by the presence of inhaled agents, such as sevoflurane, and may explain the greater vascular dysfunction observed (14). However, in our study, the clinical consequences remained minor as the intraoperative fluid volume, duration of vasopressor infusion, and lactate levels measured on postoperative day one were not significantly different.

Furthermore, we did not find an increased risk of acute renal failure. In cardiac surgery, the impact of inhaled agents on renal function remains controversial, with some studies reporting a protective action of sevoflurane and others reporting an increase in acute renal failure (14, 25). In non-cardiac surgery, sevoflurane anesthesia is sometimes associated with a risk of moderate acute renal failure (26). However, these results remain dependent on the modalities of diagnosing acute renal failure (26). In our study, using the criteria of the KDIGO group in accordance with recommendations, we did not find any impact of inhaled agents on renal function. Contrary to published data, the use of sevoflurane appears to be safe (14, 26).

Our study has several limitations.

This is a retrospective study, and some data are missing, notably the MAC of sevoflurane, as mentioned above. Other cardiac damage biomarkers, such as CK-MB or myoglobin were not assayed. However, troponin, a commonly studied marker, was deemed sufficient for assessing myocardial damage (14). Intraoperative and postoperative cardiac output data, as well as information on left ventricular ejection fraction and right heart function at discharge, are also absent. Future studies of our cardioprotective strategy should incorporate these elements to better describe the impact of total inhaled anesthesia on myocardial function.

This study was not randomized, and the allocation of patients to the two groups was based on the availability of anesthetic agents. However, they were matched using a propensity score that considered factors influencing the postoperative troponin level peak.

The scheduling of cardiac surgery patients was influenced by the health context, with more urgent and severe cases being operated on first. The mean EuroSCORE II was higher than that reported in other studies, potentially increasing the incidence of postoperative complications. However, patients in both groups were included during the same period.

Finally, this is a single-center study, and caution is needed in extrapolating the results without the support of additional large studies in other centers.

## 5 Conclusion

The use of a single device to maintain total inhaled anesthesia with sevoflurane, using the Sedaconda device, is feasible and has not been associated with an increase in postoperative complications. Instead, total inhaled anesthesia with sevoflurane appears to be an intriguing approach for improving postoperative cardiovascular outcomes. Although this strategy did not reduce the peak level of troponin in the postoperative period, the use of positive inotropic catecholamines was significantly reduced with inhaled sevoflurane anesthesia. Thus, the Sedaconda device, by facilitating the continuous administration of sevoflurane throughout intraoperative and postoperative procedures, would enable halogenated agents to express their full cardioprotective action. The role of this strategy needs further clarification.

## Data availability statement

The original contributions presented in the study are included in the article/Supplementary material, further inquiries can be directed to the corresponding author.

## Ethics statement

Ethical approval for this study was provided by our Ethical Committee of University Hospital of Toulouse (Ref RnIPH 2020-124). The studies were conducted in accordance with the local legislation and institutional requirements. Written informed consent for participation was not required from the participants or the participants' legal guardians/next of kin because this is a retrospective observational study for which French law does not require the signature of a consent form.

## Author contributions

FL: Conceptualization, Data curation, Methodology, Supervision, Validation, Writing – original draft, Writing – review & editing. PC: Data curation, Writing – original draft. ML: Investigation, Writing – review & editing. PS-V: Investigation, Writing – review & editing. AA: Visualization, Writing – original draft. BM: Supervision, Writing – review & editing. TG: Supervision, Writing – review & editing. FF: Formal analysis, Methodology, Writing – review & editing. FV-B: Validation, Writing – review & editing. VM: Conceptualization, Methodology, Supervision, Validation, Visualization, Writing – original draft, Writing – review & editing.

## References

[1] Swyers T Redford D Larson DF. Volatile anesthetic-induced preconditioning. Perfusion. (2014) 29:10–5. 10.1177/0267659113503975 24002781

[2] Steurer MP Steurer MA Baulig W Piegeler T Schläpfer M Spahn DR. Late pharmacologic conditioning with volatile anesthetics after cardiac surgery. Crit Care. (2012) 16:R191. 10.1186/cc11676 23062276 PMC3682293

[3] Uhlig C Bluth T Schwarz K Deckert S Heinrich L De Hert S. Effects of volatile anesthetics on mortality and postoperative pulmonary and other complications in patients undergoing surgery: a systematic review and meta-analysis. Anesthesiology. (2016) 124:1230–45. 10.1097/ALN.0000000000001120 27065094

[4] Landoni G Greco T Biondi-Zoccai G Nigro Neto C Febres D Pintaudi M. Anaesthetic drugs and survival: a Bayesian network meta-analysis of randomized trials in cardiac surgery. Br J Anaesth. (2013) 111:886–96. 10.1093/bja/aet231 23852263

[5] De Hert S Vlasselaers D Barbé R Ory JP Dekegel D Donnadonni R. A comparison of volatile and non volatile agents for cardioprotection during on-pump coronary surgery. Anaesthesia. (2009) 64:953–60. 10.1111/j.1365-2044.2009.06008.x 19686479

[6] Likhvantsev VV Landoni G Levikov DI Grebenchikov OA Skripkin YV Cherpakov RA. Sevoflurane versus total intravenous anesthesia for isolated coronary artery bypass surgery with cardiopulmonary bypass: a randomized trial. J Cardiothorac Vasc Anesth. (2016) 30:1221–7. 10.1053/j.jvca.2016.02.030 27431595

[7] Kunst G Klein AA. Peri-operative anaesthetic myocardial preconditioning and protection - cellular mechanisms and clinical relevance in cardiac anaesthesia. Anaesthesia. (2015) 70:467–82. 10.1111/anae.12975 25764404 PMC4402000

[8] Symons JA Myles PS. Myocardial protection with volatile anaesthetic agents during coronary artery bypass surgery: a meta-analysis. Br J Anaesth. (2006) 97:127–36. 10.1093/bja/ael149 16793778

[9] Landoni G Lomivorotov VV Nigro Neto C Monaco F Pasyuga VV Bradic N. Volatile anesthetics versus total intravenous anesthesia for cardiac surgery. N Engl J Med. (2019) 380:1214–25. 10.1056/NEJMoa1816476 30888743

[10] Baumgartner H Falk V Bax JJ De Bonis M Hamm C Holm PJ. 2017 ESC/EACTS Guidelines for the management of valvular heart disease. Eur Heart J. (2017) 38:2739–91. 10.1016/j.rec.2017.12.013 28886619

[11] Writing Committee Members Lawton JS Tamis-Holland Jacqueline E Bangalore S Bates ER Beckie TM. 2021 ACC/AHA/SCAI Guideline for Coronary Artery Revascularization. J Am Coll Cardiol. (2022) 79:e21–129. 10.1161/CIR.0000000000001039 34895950

[12] FDA Drug Shortages. Available online at: https://www.accessdata.fda.gov/scripts/drugshortages/default.cfm (accessed June 20, 2020).

[13] Labaste F Rey V Gonzalez H Marcheix B Fourcade O Minville V. AnaConDa device: solution to perform cardiac surgery without intravenous anesthetic during the corona virus disease 2019 pandemic. J Cardiothorac Vasc Anesth. (2021) 35:1267–8. 10.1053/j.jvca.2020.09.121 33082095 PMC9760556

[14] Guinot PG Ellouze O Grosjean S Berthoud V Constandache T Radhouani M. Anaesthesia and ICU sedation with sevoflurane do not reduce myocardial injury in patients undergoing cardiac surgery: A randomized prospective study. Medicine (Baltimore). (2020) 99:e23253. 10.1097/MD.0000000000023253 33327246 PMC7738139

[15] Zou B Zou F Shuster JJ Tighe PJ Koch GG Zhou H. On variance estimate for covariate adjustment by propensity score analysis. Stat Med. (2016) 35:3537–48. 10.1002/sim.6943 26999553 PMC4961520

[16] Anesthesia for Cardiac Surgery. New England Journal of Medicine. (2019). Available online at: https://www.nejm.org/doi/10.1056/NEJMc1905784 (accessed August 17, 2021).

[17] Ovize M Thibault H Przyklenk K. Myocardial conditioning: opportunities for clinical translation. Circ Res. (2013) 113:439–50. 10.1161/CIRCRESAHA.113.300764 23908331

[18] Soro M Gallego L Silva V Ballester MT Lloréns J Alvariño A. Cardioprotective effect of sevoflurane and propofol during anaesthesia and the postoperative period in coronary bypass graft surgery: a double-blind randomised study. Eur J Anaesthesiol. (2012) 29:561–9. 10.1097/EJA.0b013e3283560aea 22965457

[19] Wasowicz M Jerath A Luksun W Sharma V Mitsakakis N Meineri M. Comparison of propofol-based versus volatile-based anaesthesia and postoperative sedation in cardiac surgical patients: a prospective, randomized, study. Anaesthesiol Intensive Ther. (2018) 50:200–9. 10.5603/AIT.a2018.0012 29913033

[20] Li F Yuan Y. Meta-analysis of the cardioprotective effect of sevoflurane versus propofol during cardiac surgery. BMC Anesthesiol. (2015) 15:128. 10.1186/s12871-015-0107-8 26404434 PMC4583176

[21] Yan M Chen C Zhang F Chen G. Lidocaine abolishes the myocardial protective effect of sevoflurane post-conditioning. Acta Anaesthesiol Scand. (2008) 52:111–6. 10.1111/j.1399-6576.2007.01487.x 17996001

[22] Mio Y Bienengraeber MW Marinovic J Gutterman DD Rakic M Bosnjak ZJ. Age-related attenuation of isoflurane preconditioning in human atrial cardiomyocytes: roles for mitochondrial respiration and sarcolemmal adenosine triphosphate–sensitive potassium channel activity. Anesthesiology. (2008) 108:612–20. 10.1097/ALN.0b013e318167af2d 18362592 PMC2697447

[23] Lomivorotov VV Efremov SM Kirov MY Fominskiy EV Karaskov AM. Low-cardiac-output syndrome after cardiac surgery. J Cardiothorac Vasc Anesth. (2017) 31:291–308. 10.1053/j.jvca.2016.05.029 27671216

[24] Koponen T Karttunen J Musialowicz T Pietiläinen L Uusaro A Lahtinen P. Vasoactive-inotropic score and the prediction of morbidity and mortality after cardiac surgery. Br J Anaesth. (2019) 122:428–36. 10.1016/j.bja.2018.12.019 30857599 PMC6435836

[25] Julier K da Silva R Garcia C Bestmann L Frascarolo P Zollinger A. Preconditioning by sevoflurane decreases biochemical markers for myocardial and renal dysfunction in coronary artery bypass graft surgery: a double-blinded, placebo-controlled, multicenter study. Anesthesiology. (2003) 98:1315–27. 10.1097/00000542-200306000-00004 12766638

[26] Bang JY Lee J Oh J Song JG Hwang GS. The influence of propofol and sevoflurane on acute kidney injury after colorectal surgery: a retrospective cohort study. Anesth Analg. (2016) 123:363–70. 10.1213/ANE.0000000000001274 27088995

