# Supplementary material for: Postoperative outcomes after total sevoflurane inhalation sedation using a disposable delivery system (Sedaconda-ACD) in cardiac surgery
Source: Front Med (Lausanne). 2024 Mar 5;11:1340119. doi: 10.3389/fmed.2024.1340119 (PMC10948405; doi:10.3389/fmed.2024.1340119)
Supplement: Supplementary file 1 [file Data_Sheet_1.docx]

# Supplementary Material

Figure 1 : Standardized differences in the baseline covariates between total inhaled anesthesia with sevoflurane and intravenous anesthesia.


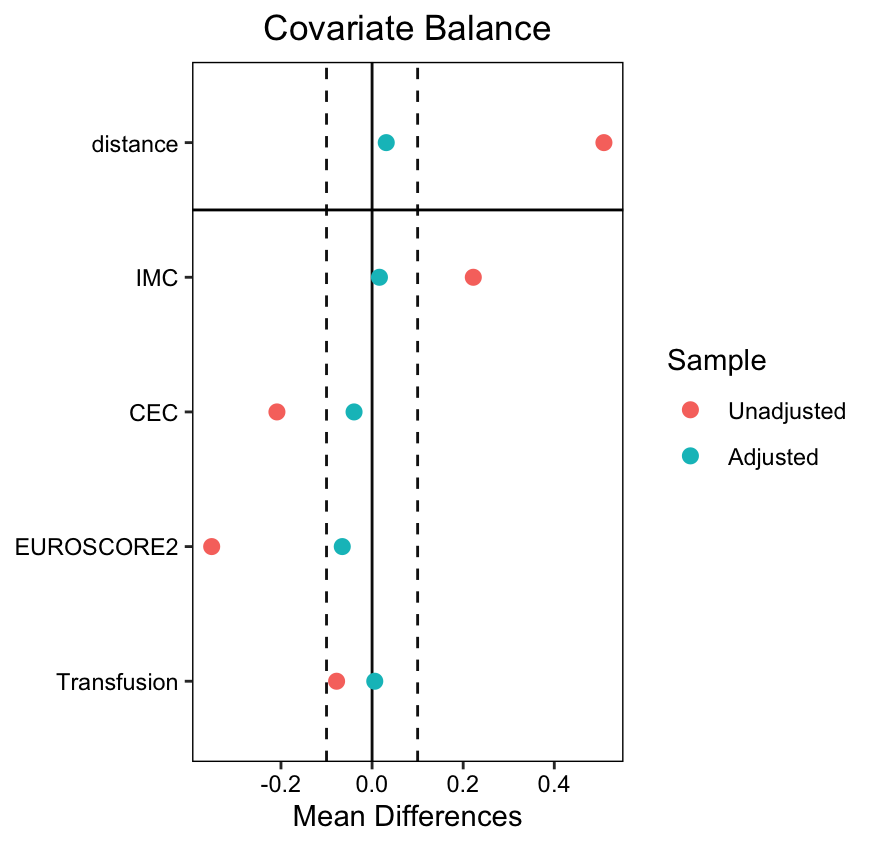


Table 1: Balance for All Data

*Std. Diff: Standardized difference, Var. Ratio: Variance ratio, eCDF: empirical cumulative distribution function*

Table 2: Balance for Matched Data

*Std. Diff: Standardized difference, Var. Ratio: Variance ratio, eCDF: empirical cumulative distribution function*

Table 3: Risk factors of postoperative inotrope administration (univariate analysis)

|  | Total  N = 654 | No Inotrop  N = 550 | Inotrop  N = 104 | p |
| --- | --- | --- | --- | --- |
| **Preoperative data** |  |  |  |  |
| Age, years | 65.5 (11.2) | 65.3 (11.3) | 66.9 (10.2) | 0.31 |
| Gender, male % (n) | 72.3 (473) | 72 (396) | 74 (77) | 0.67 |
| BMI, kg/m2 | 27.1 (4.7) | 26.9 (4.5) | 27.8 (5.2) | 0.12 |
| EuroSCORE II | 5.6 (6.4) | 4.8 (5.1) | 9.7 (10) | **< 0.0001*** |
| Coronary disease, % (n) | 48.2 (315) | 48.5 (268) | 45.2 (47) | 0.51 |
| Previous cardiac surgery, % (n) | 6.7 (44) | 5.3 (29) | 14.4 (15) | **0.0006*** |
| Chronic arterial hypertension, % (n) | 55.7 (364) | 53.6 (295) | 66.4 (69) | **0.02*** |
| Active smoking, % (n) | 2.2 (15) | 2.6 (14) | 1 (1) | 0.16 |
| Chronic occlusive arteriopathy, % (n) | 7.5 (49) | 6.9 (38) | 10.6 (11) | 0.19 |
| Diabetes (type 1 or 2), % (n) | 26 (170) | 25.8 (142) | 26.9 (28) | 0.81 |
| COPD, % (n) | 16.7 (109) | 17.3 (84) | 14.7 (25) | **0.002*** |
| Stroke, % (n) | 7.3 (48) | 6.7 (37) | 10.6 (11) | 0.25 |
| Left ventricular ejection fraction, % | 57.6 (10.9) | 58.9 (9.4) | 50.7 (14.8) | **< 0.0001*** |
| Chronic renal failure, % (n) | 16.1 (105) | 12.7 (70) | 33.7 (35) | **< 0.0001*** |
| eGFR, ml/min/1,73m2 | 77.6 (22.2) | 79.5 (21.1) | 69.2 (25.4) | **< 0.0001*** |
| Preoperative haemoglobin, g/dl | 13.6 (1.7) | 13.7 (1.7) | 13.1 (2) | **0.0006*** |
| **Peroperative data** |  |  |  |  |
| Complex surgery, % (n) | 38.7 (253) | 34.6 (190) | 60.6 (63) | **< 0.0001*** |
| Atrial fibrillation ablation | 8.4 (55) | 7.8 (43) | 11.5 (12) | 0.21 |
| Surgery duration, min | 239 (71) | 232 (63) | 276 (95) | **< 0.0001*** |
| CPB duration min | 90 (39) | 85.5 (35) | 114.4 (51) | **< 0.0001*** |
| Aortic clamping duration, min | 64.5 (30.1) | 61.4 (27.3) | 814 (37.4) | **< 0.0001** |
| Red blood transfusion, % (n) | 8.1 (53) | 5.5 (30) | 22.1 (23) | **< 0.0001*** |
| Total inhaled anesthesia, % (n) | 26 (170) | 28.4 (156) | 13.5 (14) | **0.001*** |
| **Postoperative data** | | | | |
| Lactate Day 1, mmol/l | 2.4 (1.8) | 2.15 (1.1) | 3.9 (3.4) | **< 0.0001** |
| Troponin HS, ng/l | 967.9 (1986) | 739.2 (894.3) | 2177.7 (4340) | **< 0.0001** |
| IUC LOS, days | 5.4 (4.9) | 4.9 (4.5) | 8.4 (5.9) | **< 0.0001** |
| Hospitalisation LOS, days | 12 (6.5) | 11 (5.7) | 15.4 (9.2) | **< 0.0001** |
| SPAS2 | 32.9 (9.9) | 32.2 (9.4) | 36.8 (11.3) | **0.0003** |
| Postoperative atrial fibrilation | 38.8 (254) | 36.2 (199) | 52.9 (55) | **0.001** |
| Acute renal failure | 34.3 (224) | 28.4 (156) | 65.4 (68) | **< 0.0001** |

*Quantitative data: mean (standard deviation). Comparison by Mann-Whitney test*

*Qualitative data: frequency in % (number of subjects). Comparison by Chi-square test.*

*BMI = Body Mass Index, COPD = Chronic Obstructive Pulmonary Disease, Chronic Renal Failure (GFR < 60ml/min/1.73m2), GFR: Glomerular Filtration Rate, SAPS2 = Simplified Acute Physiology Score 2, CBP: cardiopulmonary bypass*
